# Usefulness of ambulatory blood pressure measurement for hypertension management in India: the India ABPM study

**DOI:** 10.1038/s41371-019-0243-6

**Published:** 2019-09-04

**Authors:** Upendra Kaul, Priyadarshini Arambam, Srinivas Rao, Sunil Kapoor, J. P. S. Swahney, Kamal Sharma, Tiny Nair, Manoj Chopda, Jagdish Hiremath, C. K. Ponde, Abraham Oomman, B. C. Srinivas, Viraj Suvarna, Sanjiv Jasuja, Eric Borges, Willem J. Verberk

**Affiliations:** 1 Batra Heart Centre and Batra Hospital and Medical Research Centre Tughlaqabad institutional Area, New Delhi, India; 2 Care hospitals Banjara Hills and Nampally, Hyderabad, India; 3 Apollo hospitals Jubilee Hills, Hyderabad, India; 4 Sir Ganga Ram Hospital, New Delhi, India; 5 B.J. Medical College, U. N. Mehta Institute of Cardiology and Research Centre, Civil Hospital, Ahmedabad, India; 6 PRS Hospital, Department of Cardiology, Killipalam, Trivandrum, India; 7 Magnum Heart Institute, Nashik, India; 8 Ruby Hall Clinic, Pune, India; 9 Hinduja Hospital and medical research centre, Mumbai, India; 10 Apollo Hospitals, Greams Road, Chennai, India; 11 Jayadeva institute of cardiology, Bangalore, India; 12 Eris lifesciences Ltd, Ahmedabad, India; 13 Indraprastha Apollo Hospitals, Institutes of Nephrology, New Delhi, India; 14 Bombay Hospital and medical research centre, Mumbai, India; 15 CARIM School for Cardiovascular Diseases, Maastricht University, Maastricht, the Netherlands

**Keywords:** Diagnosis, Medical research

## Abstract

The present paper reports differences between office blood pressure (BP) measurement (OBPM) and ambulatory blood pressure measurement (ABPM) in a large multi-centre Indian all comers’ population visiting primary care physicians. ABPM and OBPM data from 27,472 subjects (aged 51 ± 14 years, males 68.2%, treated 45.5%) were analysed and compared. Patients were classified based on the following hypertension thresholds: systolic BP (SBP) ≥ 140 and/or diastolic BP (DBP) ≥90 mmHg for OBPM, and SBP ≥ 130 and/or DBP ≥ 80 mmHg for 24-h ABPM, and SBP ≥ 120 and/or DBP ≥ 70 mmHg for night-time ABPM and SBP ≥ 135 and/or DBP ≥ 85 mmHg for daytime ABPM, all together. White coat hypertension (WCH) was seen in 12.0% (*n* = 3304), masked hypertension (MH) in 19.3% (*n* = 5293) and 55.5% (*n* = 15,246) had sustained hypertension. Isolated night-time hypertension (INH) was diagnosed in 11.9% (*n* = 3256). Untreated subjects had MH relatively more often than treated subjects (23.0% vs. 14.8%, *p* < 0.0001; respectively). Females had higher relative risk (RR) of having WCH than males (RR 1.16 [CI 95, 1.07–1.25], *p* < 0.0001). Whereas, males had higher RR of MH than females (RR 1.09 [CI 95, 1.02–1.17] *p* < 0.01). INH subjects had lower average systolic and diastolic dipping percentages (0.7 ± 6.6/ 2.2 ± 7.9 vs. 9.0 ± 7.3/11.9 ± 8.5, *p* < 0.001) than those without INH. In conclusion, for diagnosis of hypertension there was a contradiction between OBPM and ABPM in approximately one-third of all patients, and a substantial number of patients had INH. Using ABPM in routine hypertension management can lead to a reduction in burden and associated costs for Indian healthcare.

## Introduction

In India, cardiovascular disease (CVD) is the number one cause of mortality, causing over 2 million deaths covering more than a quarter of all deaths in 2015 [1]. The fact that the age-standardised CVD death rate in India is higher than the global average [2] indicates that measures can and must be taken to stop the rising CVD burden in the future. High blood pressure (BP) is the leading CVD risk factor globally with 13% attributable deaths [3] and is causing a major increase in loss of disability-adjusted life years [4]. Improving the hypertension control rate should therefore undoubtedly lead to a reduction in CVD morbidity and mortality. Certainly, when considering that an estimated one-third of all adults are having hypertension in low- and middle-income countries [5], optimal treatment of hypertension starts with diagnosing the disease properly. Presently, diagnosis of hypertension in India is generally based on BP measurement in the clinic using a mercury sphygmomanometer, a method that can be liable to errors and misinterpretation [6]. Ambulatory BP measurement (ABPM) has proven to be a stronger predictor of all causes and cardiovascular mortality than office BP measurement (OBPM) [7]. Out-of-office BP monitoring offers specific advantages over OBPM, such as the possibility to obtain many measurements in a non-clinical setting. This has proven to reduce the white coat effect that may lead to a reduction of unnecessary treatment and thus save costs for healthcare when ambulatory [8] or self BP measurement is performed [9]. For this reason, the National Institute for Health and Care Excellence (NICE) in the UK has recommended the use of ABPM for standard clinical practice and, recently, also the JNC [10] and ESC [11] followed. In case of a high OBPM value, the patient should receive ABPM (or self-measurement of BP at home) before anti-hypertensive treatment is started [12]. Although ABPM has become a standard procedure for hypertension management in developed countries, it is not yet the case for low- and middle-income countries (LMIC). A recent survey among 260 physicians in India revealed that most physicians (72%) perform ABPM in <5% patients [13]. However, as 24-h ABPM can address major issues, such as reliably diagnosing night-time hypertension, masked hypertension (MH) and white coat hypertension (WCH), its use may lead to improved diagnosis of hypertension and reduced healthcare costs for the long-term. Therefore, the present study is aimed at investigating the prevalence of WCH, and MH and some other relevant parameters of ABPM for hypertension management in a large Indian all comers’ population visiting primary care physicians.

## Methods

### Study design and participants

A total of 32,808 patients attending 574 primary care clinics for routine management of hypertension spread throughout the whole of India participated in the present study. All patients were included between January 2017 and November 2018. OBPM was performed as usual in clinical practice without further instructions to the treating physician. Patients were referred for ABPM because their physician considered this necessary. As ABPM service was not available in the hospitals, it was made available by the sponsor Eris Lifesciences Ltd. (Ahmedabad, India) who had no further interference than providing the service. Healthcare workers visited the patient at home to connect the 24-h ABPM device to the patient and picked up the device the following day. Subsequently, The ABPM report was emailed to the treating physician. ABPM was performed according to standard instructions using validated 24-h ABPM devices (Meditech, Meditech Ltd Hungary [14] or WatchBP O3, Microlife, corporation Taiwan [15, 16]). Measurement interval times were set at 20-min intervals for 24-h. Daytime and night-time averages were based on patient’s diaries. Patient characteristics, medication intake and specific cardiovascular risk factors were registered. All subjects had their OBPM and ABPM within a period of 1 week and because physicians awaited the ABPM results, medication intake was unchanged during that period. The present study entails a post-hoc analysis with data obtained from patients visiting the hospital for routine clinical management of hypertension. Data were analysed in an anonymised way after authorisation was granted by the institutional ethics committee of the Medical Research centre (Batra Hospital, Delhi, India) from where the project was coordinated.

### Blood pressure categories

For the present study, patients were classified into a BP category based on their OBPM and ABPM values using threshold values as recommended by the European Society of Hypertension guidelines [11, 17]. BP categorisation was based on the following threshold values for OBPM and ABPM: elevated (hypertension) OBPM (SBP ≥ 140 mm Hg and/or DBP ≥ 90 mm Hg), elevated 24-h ABPM (24-h SBP ≥ 130 and/or DBP ≥ 80 mmHg), elevated daytime ABPM (daytime SBP ≥ 135 and/or DBP ≥85 mmHg) and elevated night-time ABPM as SBP ≥ 120 and/or DBP ≥ 70 mmHg).

Subjects were categorised based on OBPM and ABPM in four categories: (1) sustained normotension (SNT) normal OBPM + normal 24-h + normal daytime +  night-time BP; (2) sustained hypertension (SHT): elevated OBPM + elevated 24-h or elevated daytime or elevated night-time BP; (3) White-coat hypertension (WCH): elevated OBPM + normal 24-h + normal daytime + normal night-time BP; and (4) masked hypertension (MH): normal OBPM + elevated 24-h or elevated daytime or elevated night-time BP. Isolated night-time hypertension (INH) was defined as elevated night-time ABPM and normal daytime ABPM. Isolated daytime hypertension as normal night-time ABPM and elevated daytime ABPM.

### Patient data

Information about the subject’s age, gender, height, body weight and family history for cardiovascular diseases were collected. Also personal clinical history for cardiovascular diseases (ischaemic heart disease, myocardial infarction, heart failure, stroke, peripheral artery disease or kidney disease), presence and treatment of arterial hypertension, diabetes mellitus and dyslipidaemia were recorded. Patients were included if OBPM was performed and ABPM contained 70% or more successful readings with averages for both day and night ABPM available.

### Statistical analysis

Data analysis was performed by grouping the patients according to one of the four BP categories (SNT, SHT, WCH, MH). Given the observational nature of the study, no sample size estimation was done. All subjects included for analysis provided valid data and thus no methodology for replacing missing data was implemented. Main demographic and clinical data of the two subgroups were summarised by calculating the mean (±SD) in case of continuous variables, and the absolute (*n*) and relative (%) frequency in case of categorical variables. Differences across groups were evaluated by multi-analysis of variance (MANOVA) by entering in the analysis BP classification (SNT, SHT, WCH, MH) as independent and the parameters as represented in Table 1 as dependent variables. For isolated day and night-time hypertension the same method was used. In case of categorical variables, differences across groups were evaluated by Chi-square test. Paired *t*-tests were used to compare OBPM and ABPM values in the same participant for the full sample and within subgroups. To analyse BP values related to age, patients were divided in six age groups of equal numbers (ranks). Binary logistic regression analysis was used to determine the relative risk of gender to either masked or white coat hypertension. Results were presented in *p*-values. A *p*-value of <0.01 was considered significant. Data analysis was performed using IBM SPSS Statistics version 26 for Windows.

**Table 1:** Demographic and clinical data of 27,472 subjects included in the analysis

		Sustained normotension	Sustained hypertension	WCH	MH
	(*n* = 27,472; 10)	(*n* = 3629; 13.2%)	(*n* = 15246; 55.5%)	(*n* = 3304; 12.0)	(*n* = 5293; 19.3)
Age (years)	50.8 ± 14.4	48.4 ± 14.7	51.8 ± 14.2	48.8 ± 13.9	50.9 ± 14.8
Gender (*n*, %)					
Male	18,724 (68.2)	2335 (64.3)	10,542 (69.1)	2158 (65.3)	3689 (69.7)
Female	8748 (31.8)	1294 (35.7)	4704 (30.9)	1146 (34.7)	1604 (30.3)
Height (cm)	162.7 ± 8.3	162.6 ± 8.2	162.8 ± 8.3	162.2 ± 8.5	163.1 ± 8.1
Weight (kg)	71.6 ± 11.3	69.6 ± 11.1	72.4 ± 11.5	72.1 ± 10.9	70.4 ± 11.1
BMI (kg/m^2^)	27.1 ± 4.4	26.4 ± 4.3	27.4 ± 4.4	27.5 ± 4.3	26.5 ± 4.2
Obesity (BMI ≥30 kg/m^2^ [*n*, %])	6033 (22)	617 (17)	3667 (24.1)	807 (24.4)	942 (17.8)
Severe Obesity (BMI ≥35 kg/m^2^ [*n*, %])	1321 (4.8)	133 (3.7)	834 (5.5)	180 (5.4)	174 (3.3)
Anti-hypertensives (*n*, %)	12,495 (45.5)	1004 (27.7)	8160 (53.5)	1477 (44.7)	1854 (35)
Diabetes mellitus (*n*, %)	1968 (7.2)	168 (4.6)	1276 (8.4)	265 (8)	259 (4.9)
Dyslipidaemia	825 (3)	89 (2.5)	490 (3.2)	89 (2.7)	157 (3)
Chronic kidney disease	741 (2.7)	56 (1.5)	524 (3.4)	46 (1.4)	115 (2.2)
Family hypertension (*n*, %)	1399 (5.1)	145 (4)	853 (5.6)	173 (5.2)	228 (4.3)
Family CVE (*n*, %)	3517 (12.8)	361 (9.9)	2119 (13.9)	406 (12.3)	631 (11.9)
OSBP (mmHg)	143.8 ± 18.5	122.1 ± 11.3	153.3 ± 15.3	148.3 ± 12.4	128.3 ± 8.6
ODBP (mmHg)	86.4 ± 12.1	74.8 ± 8.9	91.4 ± 11.2	89 ± 8.8	78.4 ± 8.2
OHR (bpm)	81.8 ± 13.4	78.0 ± 13	83.3 ± 12.9	83.9 ± 12.5	78.9 ± 14.4
24-h SBP (mmHg)	131.7 ± 15.9	114.6 ± 7.4	139.5 ± 14.5	116.6 ± 6.9	130.1 ± 10.6
24-h DBP (mmHg)	78.1 ± 10.7	68.6 ± 5.9	82.3 ± 10.5	69.4 ± 6	78.1 ± 8.2
24-h HR (bpm)	75.9 ± 11	74 ± 10.2	76.8 ± 11.3	74.1 ± 10.3	76 ± 10.7
Day SBP (mmHg)	135.2 ± 15.8	118.8 ± 8.4	143 ± 14.5	121.1 ± 7.9	132.9 ± 11
Day DBP (mmHg)	81.1 ± 11.3	72 ± 7	85.2 ± 11.3	73.1 ± 7	80.5 ± 9.2
Day HR (bpm)	78.9 ± 11.8	77.3 ± 11	79.6 ± 12.1	77.4 ± 11.1	78.9 ± 11.6
Night SBP (mmHg)	124.3 ± 18	105.9 ± 7.5	132.4 ± 17	107 ± 7.3	124.4 ± 12.8
Night DBP (mmHg)	72.1 ± 11.1	61.5 ± 5.2	76.5 ± 10.8	61.8 ± 5.3	73.2 ± 8.2
Night HR (bpm)	69.8 ± 11	67.1 ± 10.2	70.9 ± 11.2	67.1 ± 10	70.1 ± 10.7
Dip SBP (%)	8 ± 7.7	10.6 ± 6	7.3 ± 7.9	11.5 ± 5.9	6.2 ± 8
Dip DBP (%)	10.8 ± 9	14.1 ± 7.3	9.8 ± 9	15.2 ± 7.1	8.6 ± 9.5
WCE SBP (mmHg)	12.1 ± 17	7.5 ± 10.2	13.8 ± 15.7	31.7 ± 13.9	−1.8 ± 11.9
WCE DBP (mmHg)	8.3 ± 11.4	6.2 ± 7.9	9.1 ± 11.1	19.6 ± 9.8	0.3 ± 8.4

The separate groups are based on office and ambulatory blood pressure measurement values. Data are shown as mean ± SD or absolute (*n*) and relative (%, in brackets) frequency. Differences across the four classification groups were all significant (*p*  < 0.0001)

*WCH* indicates white coat hypertension, *MH* masked hypertension, *BMI* body mass index, *OSBP* office systolic blood pressure, *SBP* systolic blood pressure, *DBP* diastolic blood pressure, *HR* heart rate, *bpm* beats per minute, *WCE* white coat effect (OBPM-24h ABPM). *OBPM* office blood pressure measurement, *ABPM* ambulatory blood pressure measurement

## Results

A total of 27,472 (84%) of the 32,808 subjects completed 24-h ABPM with enough successful readings (>70%) and were included for the analysis. Reasons for exclusion were because there was no OBPM value available (*n* = 235; 0.9%) or patients had <70% successful ABPM readings (*n* = 5101; 18.6%). Patients with <70% successful ABPM readings were relatively more often female (37% vs 32%), more often obese (25% vs. 22%) were less often taking medication (42% vs. 46%), had higher night-time SBP (126 vs. 124 mmHg) but lower night-time DBP (71 vs. 72 mmHg) [all *p* < 0.001]. Of the excluded patients, 3340 (65% of the excluded) had >50% but <70% successful ABPM readings (Supplement 1).

**Fig. 1:**
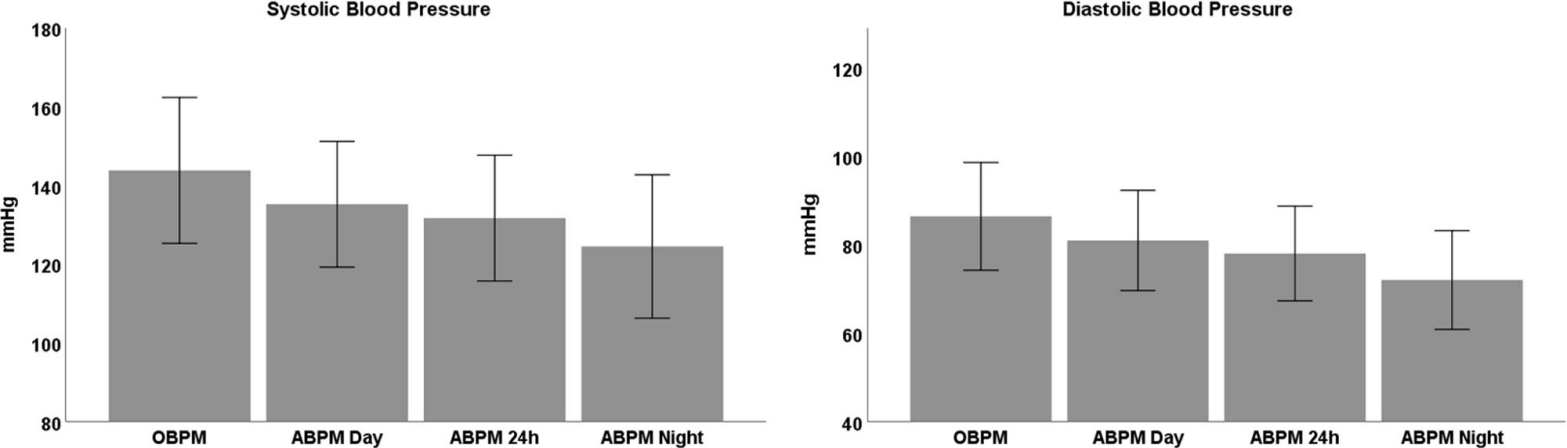
Mean blood pressure values ± SD of 27,472 patients for office blood pressure measurement (OBPM) and ambulatory blood pressure measurement (ABPM), separated for day, 24-h and night time. All values significantly differed from each other (*p* < 0.0001)

Included patients were aged 51 ± 14 years, 68.2% male and approximately half of them received antihypertensive treatment (*n* = 12,495, 45.5%). OBPM values were significantly higher than daytime ABPM, 24-h ABPM and night-time ABPM, respectively, both for SBP and DBP (Fig. 1). Based on OBPM and 24-h, daytime and night-time ABPM, patients were classified as follows: 3629 normotensives (13.2%), 15,246 sustained hypertensives (55.5%), 3304 white coat hypertensives (12.0%) and 5293 masked hypertensives (19.3%). This means that there was a contradiction between 24-h ABPM and OBPM in 8597 (31.3%) subjects.

### Blood pressure classification based on ABPM and OBPM

Anthropometric and clinical data of the participants grouped by BP classification are presented in Table 1. Subjects with SHT were older than sustained normotensives (51.8 ± 14.2 vs. 48.4 ± 14.7) more often used anti-hypertensive treatment (53.5% vs. 27.7%) and more often had diabetes (8.4% vs. 4.6%). Patients with WCH showed the highest heart rate during OBPM (83.9 ± 12.5 BPM) of all groups and the highest white coat effect based on 24-h ABPM of all groups: 31.7 ± 13.9 mmHg for SBP and 19.6 ± 9.8 mmHg for DBP. All differences were significant at *p* < 0.0001.

#### Twenty-four hour blood pressure pattern in treated and untreated patients

**Fig. 2:**
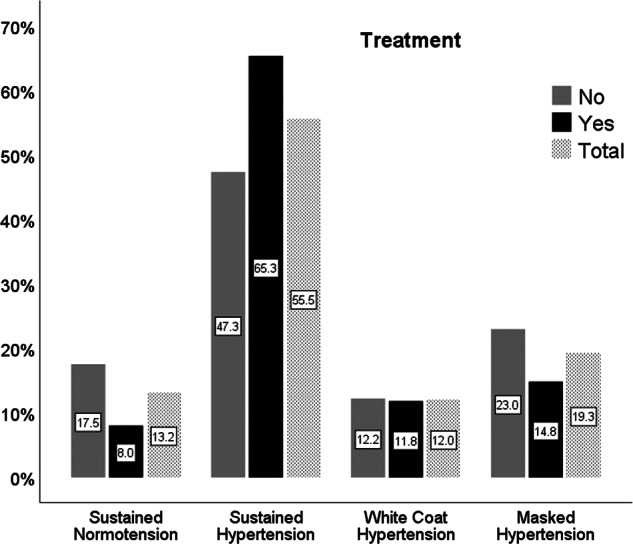
Prevalences of blood pressure classifications in percentages for patients treated and untreated for high blood pressure

Figure 2 shows BP classification prevalences between treated and untreated patients. Patients without anti-hypertensive treatment had MH relatively more often than treated subjects (23.0% vs. 14.8%, *p* < 0.0001; respectively), but there was no difference in WCH prevalence (12.2% vs.11.8%, *p* = 0.34).

In 5266 subjects (35.2%) of the untreated population, there was a contradiction in outcome between OBPM and the combination of 24-h daytime and night-time ABPM. For the treated population this contradiction was seen in 3331 subjects (26.7%), *p* < 0.0001.

### Blood pressure and gender

For OBPM females (47.6% treated) had an average higher SBP (144.3 ± 19.5 vs. 143.5 ± 18.0 mmHg, *p* < 0.0001) but lower DBP (85.2 ± 12.4 vs. 87.0 ± 12.0 *p* < 0.0001) thus a higher pulse pressure (59.1 ± 16.0 vs. 56.5 ± 14.8, *p* < 0.0001) than males (44.5% treated), but for 24-h ABPM they had similar SBP (131.6 ± 17.0 vs. 131.7 ± 15.4; *p* = 0.85) and lower DBP (76.0 ± 10.7 vs. 79.1 ± 10.5; *p* < 0.0001). Females had a higher white coat effect (OBPM-24h ABPM) than males for SBP (12.7 ± 17.3 vs. 11.8 ± 16.8, *p* < 0.0001) and DBP (9.2 ± 11.7 vs. 7.9 ± 11.3, *p* < 0.0001). This resulted in a higher relative risk (RR) of having WCH for females as compared to males (RR 1.16 [CI 95, 1.07–1.25], *p* < 0.0001). Whereas, males had a higher risk of MH as compared to females (RR 1.09 [CI 95, 1.02–1.17], *p* < 0.01).

### Blood pressure pattern related to age and gender

**Fig. 3:**
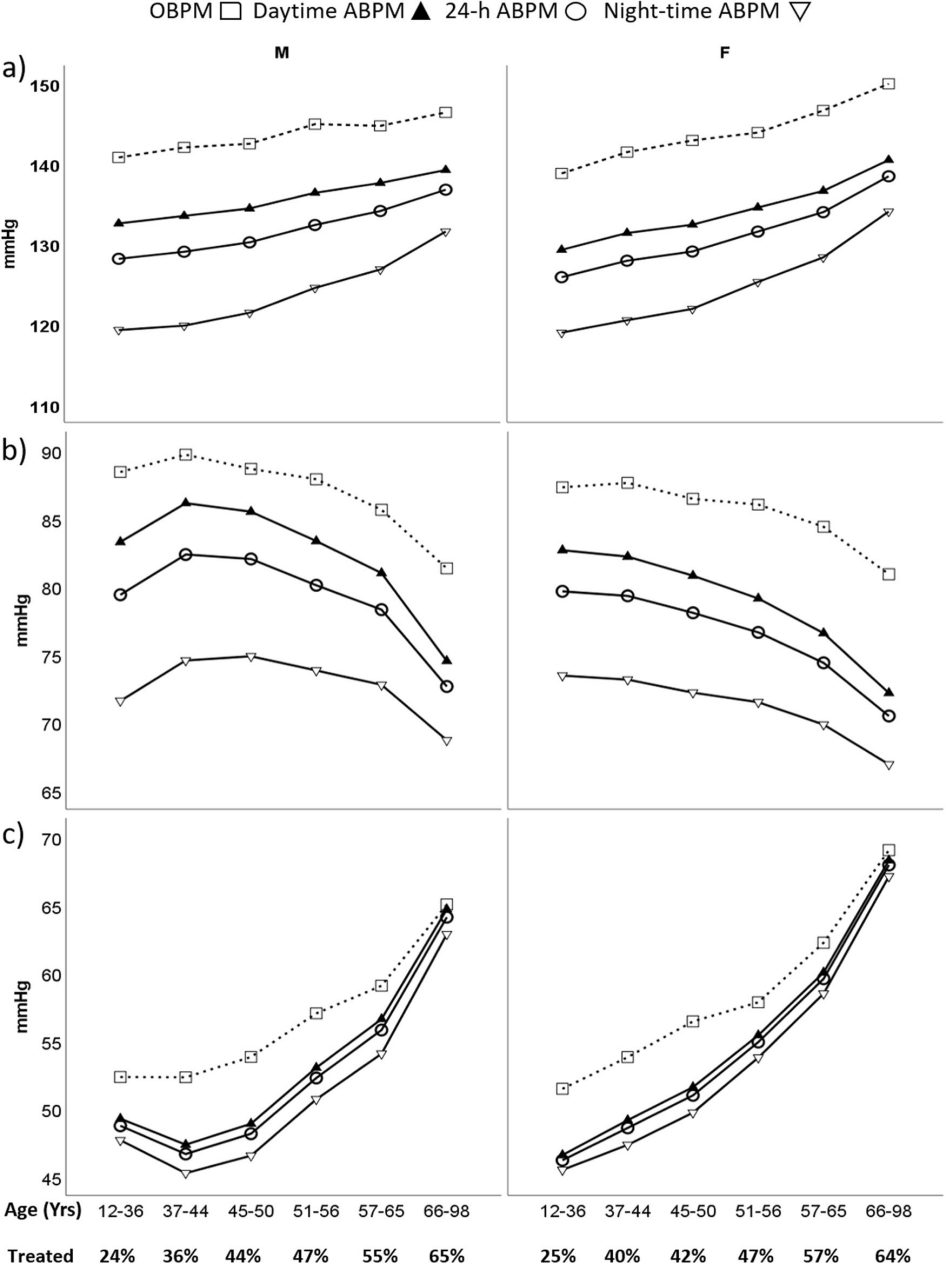
Blood pressure patterns for males (M) and females (F) separated for age categories, with the percentage patients receiving anti-hypertensive treatment given. **a** Represents systolic blood pressure, **b** diastolic blood pressure and **c** pulse pressure. OBPM, indicates office blood pressure measurement; ABPM, ambulatory blood pressure measurement

Figure 3a shows that in the first age rank (age 12 to 36 years) males had a higher office SBP and DBP (140.7 ± 15.9/88.4 ± 11.8 vs. 138.8 ± 18.1/87.3 ± 13.3; *p* < 0.001) and mean daytime and 24 h ambulatory SBP than females (128.1 ± 12.0 and 132.5 ± 12.3 vs. 125.8 ± 16.1 and 129.2 ± 16.1, respectively; *p* < 0.001), and a similar night-time ambulatory SBP (119.3 ± 13.5 and 118.9 ± 17.6; *p* = 0.51) without significant differences for DBP (Fig. 3b). For the highest age group (66 to 98 years), males had lower SBP but higher DBP than males for all ABPM values (for 24-h 136.7 ± 16.6/72.6 ± 9.5 vs.138.4 ± 17.5/70.5 ± 9.5; for daytime 139.2 ± 16.8/74.5 ± 9.9 vs.140.4 ± 17.6/72.2 ± 9.9; for night-time 131.5 ± 19.1/68.7 ± 10.6 vs. 134.0 ± 19.7/66.9 ± 10.2; all *p* < 0.001) and for office SBP (146.4 ± 19.7 vs. 149.9 ± 20.4; *p* < 0.001). For both men and women, average night-time SBP increased more with age than the average daytime ABPM value.

Figure 3b shows a clear difference in diastolic ambulatory pattern between the sexes. For males, DBP increases from young age to reach its peak at the age between 37 and 44 years. Males show a “tableau” at which DBP is stable from the age of 37 to 50 years of age, women show a continuous decrease in DBP from the age of ~35 years. After the age of 65 years, males show a steeper decrease in DBP than females but keep a higher average DBP at all age groups. Women show a steeper increase in SBP and a steeper decrease in DBP than men resulting into higher pulse pressure for OBPM and ABPM from the age of 35 years as compared to men (Fig. 3c).

### Isolated night-time hypertension based on 24-h BP measurement

Table 2 shows the number of patients with INH. In total there were 3256 subjects (11.9%) who had INH (daytime < 135/85 mmHg; night-time ≥ 120/70 mmHg). There was no difference in prevalence between the treated and untreated population (11.7% vs. 11.9%; *p* = 0.60). Women had relatively more often INH than men (14.5% vs. 10.6%, *p* < 0.0001). However, men showed isolated daytime hypertension more often than women (11.6% vs. 6.7%, *p* < 0.0001).

**Table 2:** Demographic and clinical data of 30,484 subjects included in the analysis

Total	Normal daytime and normal night-time BP (*n* = 5732; 20.9%)	Elevated daytime and elevated night-time BP (*n* = 15,727; 57.2%)	Isolated day time hypertension (*n* = 2757; 10.0%)	Isolated night-time hypertension (*n* = 3256; 11.9%)
Age (years)	49.6 ± 14.8	51.4 ± 14.1	45.9 ± 13.1	54.4 ± 15.2
Gender (*n*, %)				
Male	3557 (62.1)	11013 (70.0)	2168 (78.6)	1986 (61.0)
Female	2175 (37.9)	4714 (30.0)	589 (21.4)	1270 (39.0)
Height (cm)	162.1 ± 8.4	163 ± 8.2	163.6 ± 8.2	161.9 ± 8.3
Weight (kg)	70.4 ± 11.2	72 ± 11.4	72.9 ± 10.6	70.6 ± 11.4
BMI (kg/m^2^)	26.9 ± 4.4	27.2 ± 4.3	27.3 ± 4.2	27 ± 4.5
Obesity (BMI ≥30 kg/m^2^ [*n*, %])	1184 (20.7)	3530 (22.4)	594 (21.5)	725 (22.3)
Severe obesity (BMI ≥35 kg/m^2^ [*n*, %])	255 (4.4)	748 (4.8)	152 (5.5)	166 (5.1)
Anti-hypertensives (*n*, %)	2098 (36.6)	7948 (50.5)	982 (35.6)	1467 (45.1)
Diabetes mellitus (*n*, %)	369 (6.4)	1184 (7.5)	157 (5.7)	258 (7.9)
Dyslipidaemia	136 (2.4)	497 (3.2)	96 (3.5)	96 (2.9)
Chronic kidney disease	90 (1.6)	544 (3.5)	25 (0.9)	82 (2.5)
Family hypertension (*n*, %)	650 (11.3)	2180 (13.9)	274 (9.9)	413 (12.7)
Family CVE (*n*, %)	265 (4.6)	862 (5.5)	124 (4.5)	148 (4.5)
OSBP (mmHg)	134 ± 18.1	148.9 ± 17.6	141.2 ± 15.7	138.7 ± 16.2
ODBP (mmHg)	80.6 ± 11.6	89.3 ± 11.8	86.8 ± 10	82.3 ± 11.4
OHR (bpm)	80.3 ± 13	82.5 ± 13.6	82.7 ± 13.1	80.5 ± 13
24-h SBP (mmHg)	114.7 ± 7.3	140.6 ± 14	124.3 ± 7	124.3 ± 6.6
24-h DBP (mmHg)	67.5 ± 5.4	83.7 ± 9.8	76.5 ± 5.1	71.6 ± 5.7
24-h HR (bpm)	73.3 ± 10.3	77.1 ± 11.1	76.9 ± 10.1	74 ± 11.2
Day SBP (mmHg)	118.6 ± 8.2	144 ± 13.8	132 ± 7.7	124.7 ± 6.8
Day DBP (mmHg)	70.6 ± 6.2	86.4 ± 10.5	82.7 ± 5.4	72.2 ± 6
Day HR (bpm)	76.3 ± 11	80 ± 11.9	81.5 ± 11	75.9 ± 11.7
Night SBP (mmHg)	106.4 ± 7.7	133.7 ± 16.5	108.7 ± 6.4	123.7 ± 8.9
Night DBP (mmHg)	60.9 ± 5.4	77.9 ± 10	64.1 ± 4.5	70.4 ± 6.5
Night HR (bpm)	66.9 ± 10.3	71.2 ± 11	67.8 ± 9.6	70 ± 11.6
Dip SBP (%, SD)	10.1 (5.9)	7.1 (7)	17.5 (4.7)	0.7 (6.6)
Dip DBP (%, SD)	13.4 (7.1)	9.6 (7.9)	22.4 (5.3)	2.2 (7.9)
WCE SBP (mmHg)	19.3 ± 17.6	8.2 ± 16.2	16.9 ± 15	14.3 ± 16.0
WCE DBP (mmHg)	13.2 ± 11.4	5.7 ± 10.9	10.3 ± 10.0	10.7 ± 11.4

The separate groups are based on daytime and night-time ambulatory blood pressure (BP) measurement values. Data are shown as mean ± SD or absolute (*n*) and relative (%, in brackets) frequency. Differences across the four groups were all significant (*p* < 0.0001)

*SBP* systolic blood pressure, *DBP* diastolic BP, *HR* heart rate, *Dip* dipping percentage, *WCE* white coat effect (Office BP-24 h BP), *NT* normotension, *HT* hypertension, *MH* masked hypertension, *WCH* white coat hypertension

Patients with INH had similar night-time (123.7 ± 8.9/ 70.4 ± 6.5 vs. 124.4 ± 18.9/ 72.3 ± 11.5) but lower daytime ABPM SBP and DBP values (136.6 ± 16.2/82.3 ±11.3 vs.124.76.8 /72.2±6.0 mmHg, *p* < 0.001), thus a low dipping percentage (0.7 ± 6.6% vs. 9.0 ± 7.3%, *p* < 0.001) and were on average older (54.4 ± 15.2 vs. 50.3 ± 14.3 years, *p* < 0.001) than those without INH.

## Discussion

To the best of our knowledge, this is the first large population study demonstrating the importance of 24-ABPM for hypertension management in general healthcare in India. Considering that 24-h ABPM is superior to OBPM for diagnosing hypertension, the present study showed that from the 27,472 patients, 8597 patients (31.3%) would have been wrongly diagnosed if only (conventional) OBPM was considered as common in current Indian primary care practice. For patients without treatment, this misclassification percentage was higher than in those who were treated (35.2% vs. 26.7%; *p* < 0.0001). The high number of patients with MH (*n* = 3439 (23.0%)) in the untreated group is concerning, as these patients would not receive anti-hypertensive treatment if only OBPM were performed, whereas their cardiovascular risk is similar to the patients with hypertension [18]. In addition, there were 3256 subjects (11.9%) who had INH and its prevalence among women was relatively higher than for men with 14.5% vs. 10.6%, respectively.

### Strength and limitations of the study

The strength of the present study lies in the large number of subjects and the fact that there was an equal distribution of treated and untreated patients receiving both OBPM and ABPM in a short-time frame. However, this study should also be seen within the context of its limitations; there were more males than females participating in the present study (68% vs 32%). This higher male prevalence may have had some influence on the outcome due to significant differences related to gender such as, e.g., the higher prevalence of INH and a higher systolic white coat effect in women as was found in the present study. In addition, treating physicians were not instructed how to perform OBPM but they performed the procedure according to routine practice. This means that there probably were differences in OBPM performance regarding the device (brand) that was used, technique (oscillometric, aneroid or mercury) and number of measurements. On the other hand, this represents general clinical practice and how clinic BP measurement is performed in India and it may possibly reveal the limitations of this standard procedure. The prevalence of WCH and MH of the present study is based on a single OBPM visit, which could lead to erroneous diagnosis in real practice in case of different outcome as compared to ABPM. However, possibly some physicians, who normally do not use ABPM, base their diagnosis and/or treatment on more than one OBPM per visit and/or on more than one clinical visit. This most likely gives a better estimation of the patient’s BP and would lead to a lower rate of erroneous diagnosis. Taking the average BP from multiple clinical visits is more in line with hypertension management guidelines, but obviously these seem to have a low adherence rate in clinical practice in India [19]. The subjects analysed in the present study were selected by their treating physicians to undergo ABPM, which may have caused a selection bias. For example, ABPM may have been requested because the obtained OBPM values were close to threshold values or not what the physician expected. The fact that <20% of the treated patients had normal 24-h and daytime and night-time values, which was <30% in the untreated population, suggests possible selection bias. For the current study, we included patients with >70% successful readings as recommended in the guidelines [20]. However, this inclusion criterium might have contributed to a selection bias. Excluded patients were more often female, untreated, obese and had higher night-time BP. The latter indicates that the subjects might have experienced the procedure as more inconvenient during sleep or it disturbed their sleep because higher cuff inflation pressure was needed for BP measurement. A higher rate of obesity also suggests more cuff inflation pressure was needed for measurement due to higher arm-circumferences, which causes more inconvenience. Finally, the high number of patients with insufficient readings suggests that the patient instruction procedure could have been better.

### Office blood pressure measurement (OBPM)

The prevalence of white coat and masked hypertension together of 31% is similar to another large ABPM data registry that contains ABPM and OBPM data of >14,000 adult patients around the globe [21]. Although that study showed a higher prevalence of white coat (23% vs. 12%) and a lower prevalence of masked hypertensives (10% vs. 19%, respectively) as compared to the present study. However, this difference in prevalence may not be directly related to the study population, but to the method of calculating the WCH and MH prevalences. In the present study, it was chosen to consider daytime, 24‐h, and night-time average BPs all together, which leads to a lower prevalence of WCH and a higher prevalence of MH than when considering only the 24-h ABPM average alone as was used in the ABPM data registry [22]. Considering only 24-h ABPM average for the present study would have led to more comparable prevalences for WCH and MH of 20% and 13%, respectively. However, using the average of 24-h ABPM also causes a risk to miss elevated nocturnal blood pressure, which is the most important cardiovascular risk predictor [23]. The average BP obtained from less frequent night-time measurements could be diluted by the (normal) average BP obtained from the higher number of daytime measurements, leading to a normal 24-h average BP [22]. Nevertheless, the prevalence of MH in the present study is relatively high as compared to other studies, which may be additionally explained by other factors such as the higher male prevalence and the fact that treated patients showed a higher prevalence of MH than untreated patients, which is in contradiction with earlier findings [18]. The latter may be a consequence of the potential patient selection bias as previously mentioned. In addition, digit preference (Supplement 2) and the way OBPM is usually performed in Indian clinics may have contributed to the high MH prevalence (Supplement 3).

### Differences between sexes regarding physician visits

The present data show a male to female ratio of 2:1. As the data are obtained from clinical practice, this suggests that females are less likely to visit a doctor and those who visit a doctor are on average 2 years older than males. Although the present study showed that females were relatively more often sustained normotensive (14.8% vs. 12.5%) and less often sustained hypertensive than males (53.8% vs. 56.3%), it is unlikely that the lower cardiovascular risk (cardiovascular events in India) justifies these large differences. Although women have lower BP at younger age, this rapidly changes after menopause. Considering the fact that women have a higher life expectancy than men in India [24] one would expect more females in need for hypertension treatment at older age. Therefore, the underrepresentation of females in GP and hospital visits most likely is a cultural problem [25] in need for an urgent change.

### Isolated night-time hypertension

The definition of WCH and MH is often based on OBPM and (awake) daytime ABPM [26] although 24-h ABPM is also common [27]. The present study clearly supports the use of combining all ABPM average of 24-h, daytime and night-time, and showed that it is often not enough to look at the daytime ABPM alone as ~12% of all patients had INH, and this prevalence increases with age. Of those with INH who were untreated, ~72% had normal 24-h ABPM values. These patients generally were recognised by the absence of BP decrease during night as compared to daytime BP. Studies have shown that also normotensive subjects with a non-dipper BP profile have increased left ventricular mass and relative wall thickness, reduced myocardial diastolic function, increased urinary albumin excretion, increased prevalence of diabetic retinopathy, and impaired glucose tolerance [28]. This indicates that treatment based on the average of 24-h ABPM alone may not be enough. Especially, when considering that elevated night-time BP is a better predictor than daytime BP in predicting fatal cardiovascular events [29] and is related to high cardiovascular risk, independently of either clinic or daytime ABPM [30]. For this group, hypertension may also remain undiagnosed by self-BP measurement at home unless a self-measurement device with the possibility to measure BP at night would be used [31]. However, if patients are already diagnosed with hypertension and receive anti-hypertensive treatment, night-time BP provides important information for optimising anti-hypertensive treatment. For instance, these patients might benefit from taking their drugs in the evening or having medication at both day and night-time.

### Clinical characteristics

Some studies found typical patient characteristics linked to either WCH (e.g., more likely to be female [32], high BMI [33]) or MH, e.g., smokers [18]. Results from the present study showed that females had a higher risk of INH and a higher systolic white coat effect than males, whereas males more often had MH. In addition, patients with WCH were more likely to have a higher heart rate during OBPM.

### Masked hypertension

In 2011, NICE in the UK implemented the use of ABPM in their guidelines “Hypertension in adults: diagnosis and management” [12]. The recommendation applies to cases where OBPM is 140/90 mmHg or higher as this could help to filter out cases of WCH and, therefore, reduce healthcare costs because of reduction in unnecessary treatment [8]. Unfortunately, this recommendation does not cover cases of MH. However, in contradiction to what is often believed it cannot be assumed that OBPM is lower than ABPM [34] so that patients with normal OBPM may benefit from additional ABPM. Standard ABPM performance in all subjects might seem costly and a logistically inconvenient procedure. However, as patients with MH have similar risks as those with hypertension [35], their treatment may lead to an overall reduction of cardiovascular events and thus in reduced burden for healthcare.

### ABPM for clinical healthcare

The present study also reveals that over 80% of treated patients have uncontrolled hypertension based on ABPM (Supplement 4), and thus emphasises the importance of proper BP measurement after hypertension has been diagnosed. The fact that 31% of the OBPM results in the present study differ from ABPM for diagnosing hypertension, together with the overwhelming evidence of ABPM’s superiority over OBPM as a cardiovascular risk predictor, pleads for the use of ABPM in standard hypertension management [36]. This statement is supported in the latest guidelines from both the European [11] and American [10] cardiovascular authorities.

The use of ABPM in hypertension management has proven its value in India not only for adults but also showed to be beneficial for use among children. In a paediatric clinic in India [37], ABPM was performed in children with incidental high OBPM, chronic kidney disease, renal transplant, solitary kidney, renal transplant or post-op for co-arction of the aorta. This resulted in the finding of WCH and MH in 27% and 21% of cases, respectively. In addition, ABPM resulted in change of management in 26% of cases, which supports the use of ABPM in paediatric care.

## Conclusion

The present study demonstrated convincing evidence regarding the usefulness of ABPM for standard hypertension management in India. Using ABPM may prevent misdiagnosis in approximately one-third of all treated and untreated subjects. This could reduce unnecessary treatment because of WCH and lead to improved treatment for those with MH and/or INH.

## Summary

### What is known about this topic

- There is often discrepancy between office blood pressure measurement and ambulatory blood pressure measurement; white coat hypertension (WCH) and masked hypertension (MH).
- Ambulatory blood pressure measurement is superior to office blood pressure measurement and therefore recommended for hypertension management.
- Night-time blood pressure measurement is an important cardiovascular risk predictor.

### What this study adds

- This is the first largescale study performed comparing OBPM and ABPM in the whole of India. Certain parameters can be specific for certain populations but also the hypertension management method can be country specific.
- WCH was seen in 12% of the population and 19% had MH. Females had higher risks of WCH and men were at higher risk of having MH.
- Isolated night-time hypertension was diagnosed in 12% of the study subjects and provide an extra reason to perform ABPM in Indian healthcare in order to improve overall hypertension management.

## Supplementary information

Supplement 1

Supplement 2

Supplement 3

Supplement 4
