# Supplementary material for: Usefulness of ambulatory blood pressure measurement for hypertension management in India: the India ABPM study
Source: J Hum Hypertens. 2019 Sep 4;34(6):457–67. doi: 10.1038/s41371-019-0243-6 (PMC7299842; doi:10.1038/s41371-019-0243-6)
Supplement: Supplementary file 1 — Supplement 1 [file 41371_2019_243_MOESM1_ESM.docx]

**Supplement 1, characteristics of included (2.00) and excluded (.00 and 1.00) patients.**


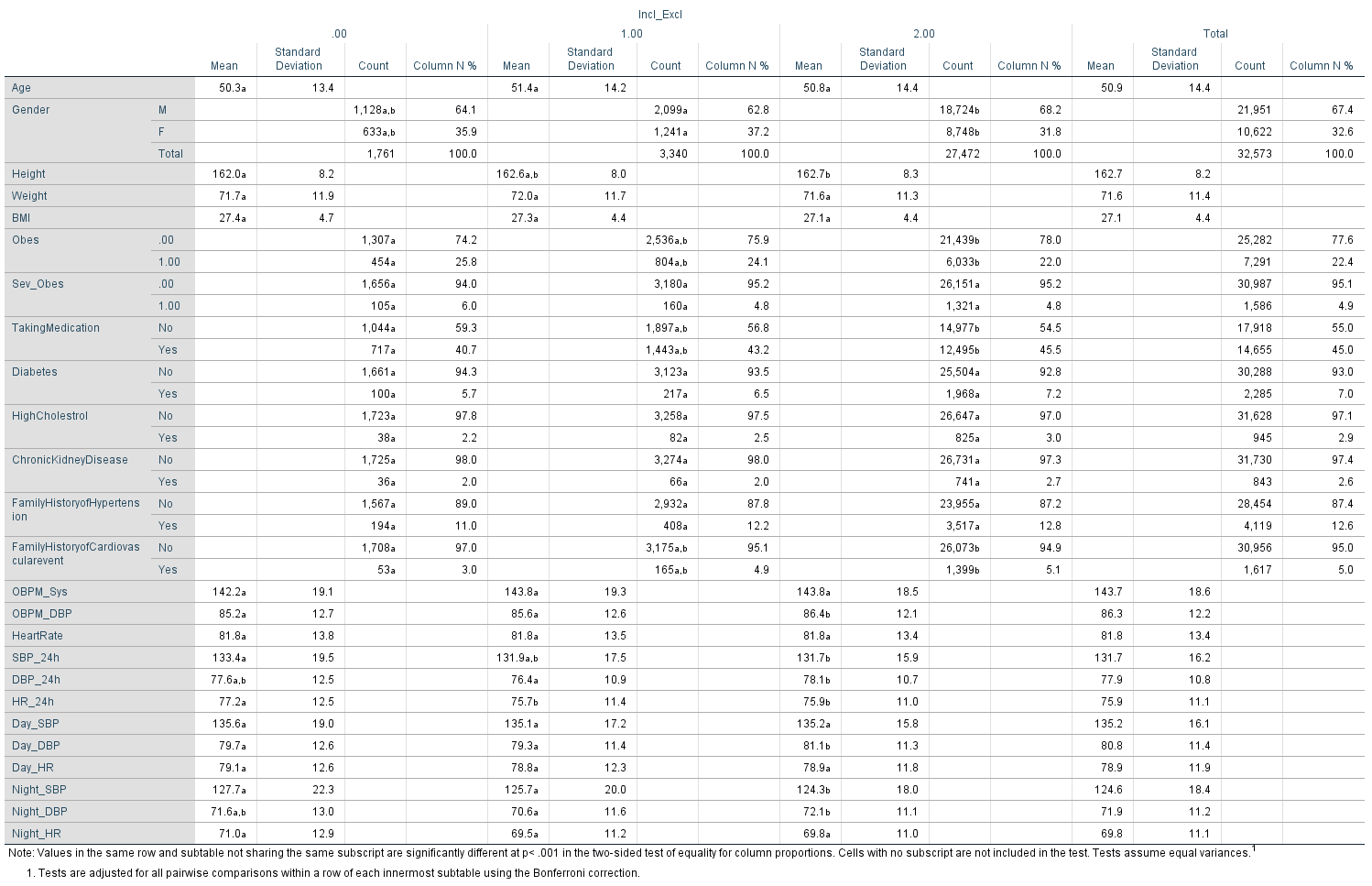


“.00” indicates less than 50% successful Ambulatory blood pressure readings; “1.00”, between 50 and 70% successful readings; “2.00”, 70% or more successful readings.
