# Supplementary figures and images for: Usefulness of ambulatory blood pressure measurement for hypertension management in India: the India ABPM study
Source: J Hum Hypertens. 2019 Sep 4;34(6):457–67. doi: 10.1038/s41371-019-0243-6 (PMC7299842; doi:10.1038/s41371-019-0243-6)

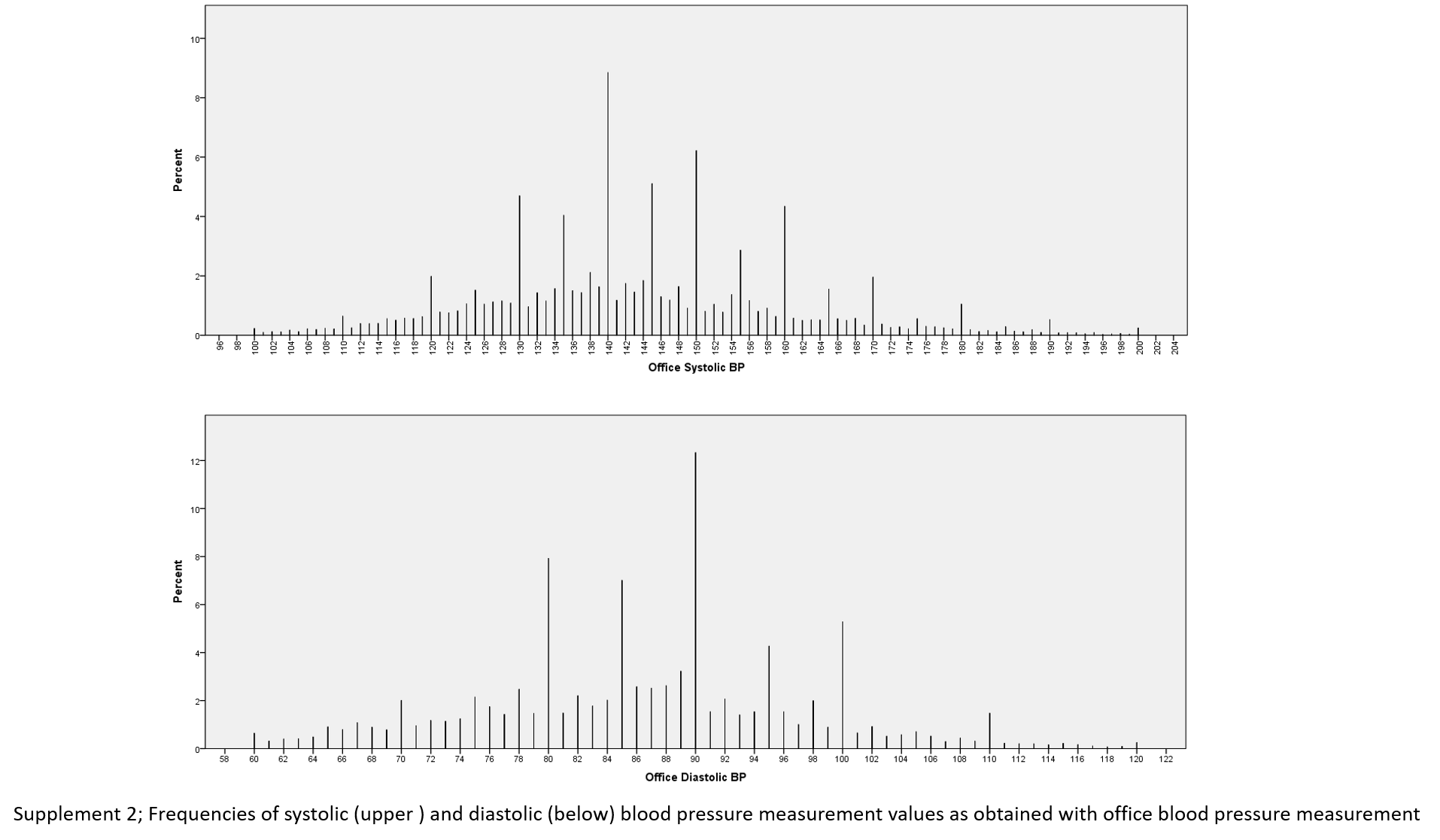

Supplement: Supplementary file 2 — Supplement 2 [file 41371_2019_243_MOESM2_ESM.tif]
