# Supplementary material for: Usefulness of ambulatory blood pressure measurement for hypertension management in India: the India ABPM study
Source: J Hum Hypertens. 2019 Sep 4;34(6):457–67. doi: 10.1038/s41371-019-0243-6 (PMC7299842; doi:10.1038/s41371-019-0243-6)
Supplement: Supplementary file 3 — Supplement 3 [file 41371_2019_243_MOESM3_ESM.docx]

**Office blood pressure measurement in Indian General practice.**

A prospective, observational study to current practices of OBPM among 400 medical and paramedical staff working in various hospitals of Northern India found that that only 1.47% (3 doctors) had accurate practice based on AHA guidelines and among paramedical staff only 0.5% (1 nurse) had accurate practice. For example, only 42% of the doctors and 30% of the nurses knew that 2 BP measurements need to be taken when measuring in the clinic ^1^. In addition, as doctors in India barely spent 2 minutes seeing their patients it is most unlikely that more than one measurement is performed ^2^. Taking only one reading during OBPM may have led to an overestimated white coat prevalence as the second measurement is usually lower than the first ^3^. Since December 2017 mercury is officially banned in India but this only means that physicians are not allowed to purchase new mercury devices. These instruments are still available in most parts of India so that most physicians still use mercury devices for OBPM ^4^. Also, for the present study it is estimated that 70% of all doctors used mercury devices for OBPM and approximately 30% used digital blood pressure monitors. A disadvantage of manual blood pressure monitors (Mercury and aneroid) is that these need frequent calibration which is often not performed ^1^ and therefore may lead to under-reading high blood pressure values. This would lead to an increase in the prevalence of MH. However, digit preference seems the biggest cause of error in manual blood pressure measurement in general and also in the present study. A separate analysis of the OBPM data showed a clear increase in measurement frequency for rounding to the nearest 0 and 5 for both systolic and diastolic blood pressure measurement with peaks at 140 mmHg (n=2,697; 8.9%) and 90 mmHg (n=3,760; 12.8%) for systolic and diastolic blood pressure, respectively (supplement 2). In total there were 5,623 subjects (18.4%) who, without using ABPM, would have been diagnosed with hypertension based on OBPM values of 140 mmHg systolic and/or 90 mmHg diastolic. Of these subjects 40% appeared to have normal ABPM values. These results indicate that digit preference plays an important role in erroneous BP measurement and pleads for the use of automated blood pressure monitor in clinical practice ^5^. An additional value of using digital monitors is that heart rate is automatically determined which might add useful information as the present study showed that patients with WCH often had a high heart rate during OBPM.

1. Mohan B, Aslam N, Ralhan U, Sharma S, Gupta N, Singh VP, Takkar S, Wander GS. Office blood pressure measurement practices among community health providers (medical and paramedical) in northern district of India. *Indian Heart J* 2014; 66**:** 401-407.

2. Irving G, Neves AL, Dambha-Miller H, Oishi A, Tagashira H, Verho A, Holden J. International variations in primary care physician consultation time: a systematic review of 67 countries. *BMJ Open* 2017; 7**:** e017902.

3. Handler J, Zhao Y, Egan BM. Impact of the number of blood pressure measurements on blood pressure classification in US adults: NHANES 1999-2008. *J Clin Hypertens (Greenwich)* 2012; 14**:** 751-759.

4. Wander GS, Ram CVS. Blood pressure - Methods to record & numbers that are significant: Lets make a tailored suit to suit us. *Indian J Med Res* 2018; 147**:** 435-438.

5. O'Brien E, Asmar R, Beilin L, Imai Y, Mancia G, Mengden T, Myers M, Padfield P, Palatini P, Parati G, Pickering T, Redon J, Staessen J, Stergiou G, Verdecchia P, European Society of Hypertension Working Group on Blood Pressure M. Practice guidelines of the European Society of Hypertension for clinic, ambulatory and self blood pressure measurement. *J Hypertens* 2005; 23**:** 697-701.
