# Supplementary material for: Usefulness of ambulatory blood pressure measurement for hypertension management in India: the India ABPM study
Source: J Hum Hypertens. 2019 Sep 4;34(6):457–67. doi: 10.1038/s41371-019-0243-6 (PMC7299842; doi:10.1038/s41371-019-0243-6)
Supplement: Supplementary file 4 — Supplement 4 [file 41371_2019_243_MOESM4_ESM.docx]

**
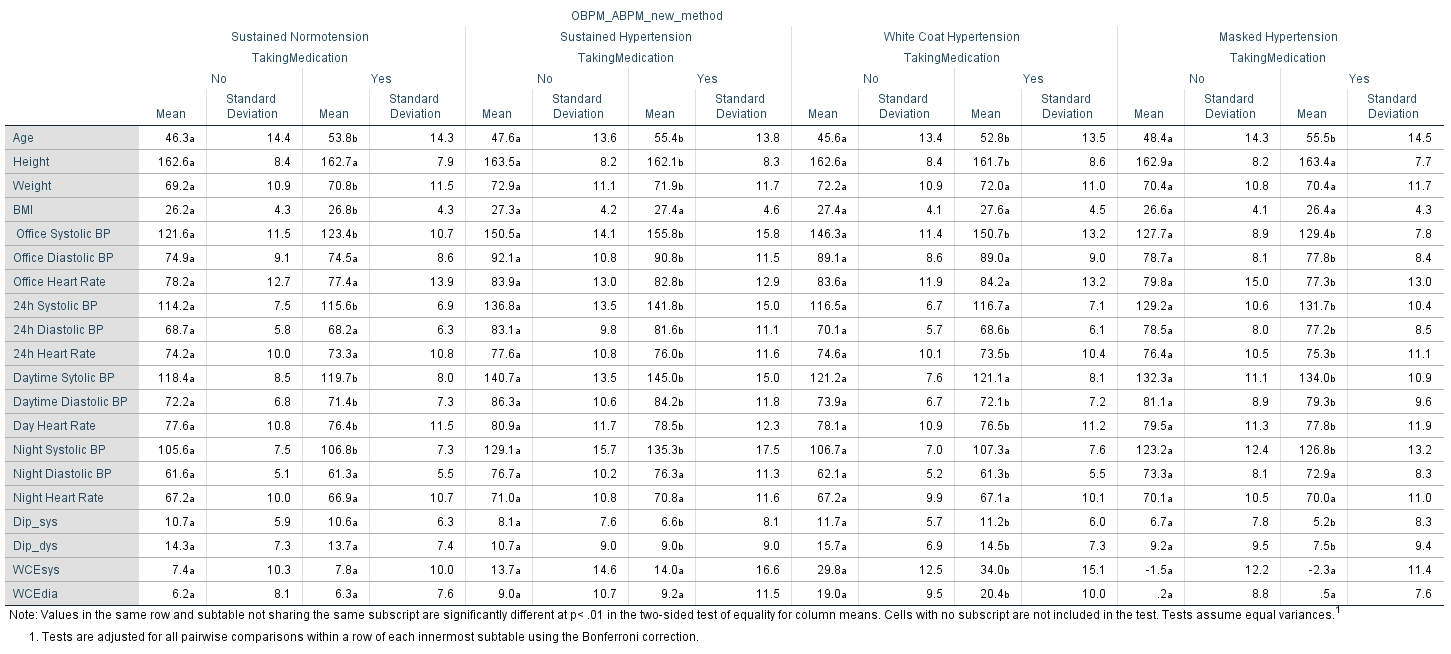
**

**Supplement 4a; characteristics of subjects separated by treated and untreated for hypertension (Average±SD values )**


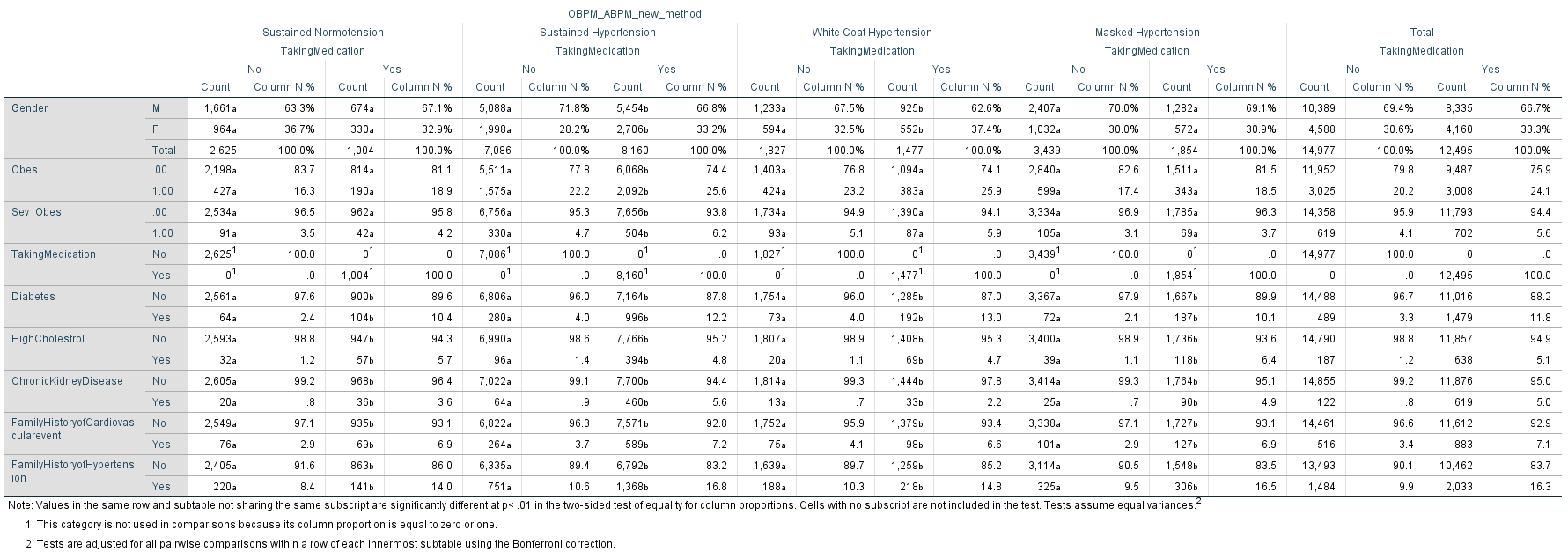


**Supplement 4b; characteristics of subjects (categorical variables) separated by treated and untreated for hypertension (number and % within groups [column] )**
